# Young people's experiences of setting and monitoring goals in school‐based counselling: A thematic analysis

**DOI:** 10.1111/papt.12581

**Published:** 2025-02-24

**Authors:** Charlie Duncan, Jacqueline Hayes, Mick Cooper

**Affiliations:** ^1^ University of Roehampton London UK; ^2^ British Association for Counselling and Psychotherapy Lutterworth UK; ^3^ Independent Researcher‐Practitioner Lewes UK

**Keywords:** experiences, monitoring goals, School‐based counselling, setting goals, thematic analysis

## Abstract

**Objective:**

To understand young people's experiences of setting and monitoring goals in the context of school‐based counselling.

**Design:**

Qualitative interview study of young people aged 13–16 years old who had undertaken school‐based counselling and who had explicitly set and monitored goals.

**Methods:**

Nineteen young people who were predominantly female (89.5%) and around half of whom were of white/European and/or British ethnicity (52.6%) were recruited from 4 secondary schools in London, UK. A reflexive thematic analysis was undertaken to identify themes.

**Results:**

Fourteen themes were identified, which reflected both helpful and unhelpful aspects of working with goals. For some young people, goals were motivating, provided a tangible representation of progress, and focused the therapeutic work. For others, goals could mirror a sense of “stuckness” and elicit negative emotions when not progressed towards in a linear fashion. Assigning a number to goal progress meant that some young people felt it did not fully capture the context of their experience, although some did find this practice helpful. Similarly, not all young people found it helpful to monitor progress at every session.

**Conclusions:**

Our findings align with the wider adult literature in that experiences of working with goals are mixed. Recommendations for practice include offering choice in the frequency and way goal progress is monitored, and using clinical judgement when working with goals. This might include noticing when goal setting or monitoring is contributing to young people's feelings of low self‐worth  and adjusting practice accordingly.

## INTRODUCTION

Global estimates suggest that 14%, or 1 in 7, 10–19 year olds experience a mental health disorder (WHO, 2021). In England alone these estimates are even greater, with approximately 1 in 5 children and young people aged 8–25 years old estimated to have a “probable mental disorder” in 2023 (NHS Digital, 2023). Whilst statutorily funded treatments for mental health issues are available to children and young people in England through the NHS‐funded Child and Adolescent Mental Health Services (CAMHS), these services often have long waiting times, particularly in circumstances where a young person is assessed as being “less severe” (Edbrooke‐Childs & Deighton, 2020). School‐based counselling is an alternative intervention that is commonly available to young people, does not have severity thresholds for entry, is easily accessible, and generally operates with a short waiting list (Cooper, 2013; Copeland et al., 2023). However, evidence on the effectiveness of school‐based counselling in the UK, which typically follows a person‐centred/humanistic model, is mixed (Cooper et al., 2021; Copeland et al., 2023).

Goals have been defined as “internal representations of desired states, where states are broadly construed as outcomes, events, or processes” (Austin & Vancouver, 1996; p. 338). Goals can be understood as aspirations, hopes, or outcomes that people strive for. In the context of a psychotherapeutic intervention, such as school‐based counselling, explicit goal setting typically takes place in the initial sessions as a collaborative exercise between therapist and client (Jacob et al., 2022).

The monitoring of goal progress has been recommended by professional therapeutic organisations. For instance, the British Association for Counselling and Psychotherapy (BACP) state in their competency framework that counsellors should have “the ability to record the child/young person's identified goals for counselling, with the aim of evaluating whether they have been met by review dates or at the end of counselling” (BACP, 2022; p. 88). One means of achieving this is using the Goal‐Based Outcome (GBO) tool (Law & Jacob, 2015). The GBO tool allows up to three personal goals to be set, either by the client or collaboratively between client, counsellor and/or caregiver, with progress rated on a scale from 0 (“not met at all”) to 10 (“completely met”). Progress ratings can be taken at any interval, including pre‐ and post‐therapy or at every session.

Best practice recommendations encourage flexibility around goal setting, advising that new goals may emerge as the therapeutic work progresses, or goals that were an initial focus may subsequently become redundant—either because they have already been attained or because they are no longer a priority for the client (BACP, 2022; Jacob et al., 2022). Existing research suggests that common goal types young people set when accessing mental health services include “goals targeting specific issues, symptoms, emotions, and behaviours”, “interpersonal goals”, “personal growth goals”, and goals related to “returning to, and engaging in, activities” (Mok et al., 2024). In school‐based counselling services, common specific goals include: “increasing self‐confidence and self‐acceptance”, “controlling or reducing anger”, “increasing feelings of happiness” and “improving relationships with family” (Rupani et al., 2013).

The GBO tool is an example of an idiographic patient reported outcome measure (I‐PROM) whereby the items are generated by clients (e.g. the goal set is defined by the client, rather than it being predetermined), yet the scoring follows a standardised format. This is in contrast to nomothetic PROMs which do not have any individualised elements. Research indicates that adult clients perceive several benefits to using I‐PROMs in therapy, including facilitating self‐reflection, empowering clients by involving them in the treatment process, and increasing client commitment to therapy through an enhanced sense of responsibility (Solstad et al., 2023). There are also indications that I‐PROMS, and specifically the GBO tool, are more sensitive measures of individual change than standardised nomothetic measures (Duncan et al., 2023; Edbrooke‐Childs et al., 2015). However, adult clients also perceive several drawbacks to the use of I‐PROMs. A recent systematic review found that unhelpful aspects of I‐PROMs included feeling demotivated from a lack of progress and, specific to working with goals, monitoring goals that were no longer relevant (Solstad et al., 2023). Di Malta et al. (2019) explored adult clients' experiences of goal‐oriented practices, as well as the relationship between attitudes towards goals and outcomes. They identified several benefits of goal‐oriented practices including facilitating self‐awareness, enhancing motivation, providing a consistent and predictable structure to the therapeutic work, and reinforcing client progress. However, they also found clear evidence that some clients also found the practice challenging in terms of difficulties formulating goals, feeling demotivated, increasing a fear of failure, and increasing negative feelings if little or no progress was made.

There is less evidence available on young people's views on, and experiences of, both nomothetic and idiographic PROMs. Given this, it is pertinent to consider the literature on their experiences of PROMs more broadly than simply I‐PROMs. Existing evidence suggests that young people experience several benefits to using PROMs including their ability to reflect their feelings to their practitioner as an additional form of communication, the empowerment that develops through tracking one's own progress, and the ability measures offer young people to reflect on their emotions (Demkowicz et al., 2020; Stasiak et al., 2013; Wolpert et al., 2016). However, young people have also expressed concerns that responding negatively might impact on the relationship with their practitioner, narrow their access to care, or not capture their own unique experience (Stasiak et al., 2013; Wolpert et al., 2016). There is also some evidence that young people can find the lack of progress, or low scores on PROMs, to be demoralising, and that the response options provided cannot capture the context around reasons given for response ratings (Demkowicz et al., 2020; Stasiak et al., 2013). Finally, some young people have expressed their concerns around using PROMs from the outset, and instead preferred to use their initial sessions to build a relationship with their practitioner (Wolpert et al., 2016).

Even more limited is the available literature on young people's experiences of working with a goal measure in psychological therapy. A 2015 study which explored young people's feedback on the use of the Goal Attainment Scale (GAS; Kiresuk et al., 1994) in a mental health setting found that young people reported using the tool to be “motivating”, useful as a tangible representation of goals and progress, and helpful as a way of devising measurable, realistic goals (Cairns et al., 2015). However, less is understood about the mechanisms of goal setting with young people. A recent review found that collaborative goal setting could facilitate trusting relationships between young people and therapists by “helping young people to feel understood, valued and that practitioners are listening to them” (Jacob et al., 2022; p. 4). Furthermore, a small‐scale, qualitative study undertaken in New Zealand asked nine 16–24 year olds about their experiences of using goals within psychological therapy (Penno et al., 2022). They found mixed experiences of the helpfulness of the process, with some young people describing it as “motivating”, and experiencing positive emotions when goals were progressed towards or attained. Conversely, some young people expressed fears around goal setting and negative emotions when goals were not progressed towards, which they sometimes experienced as a personal failure. The authors noted that the limitations of the study lay in the relatively small sample size, a lack of recorded demographic data, and that young people were at different stages–and experiencing different modalities–of therapy (Penno et al., 2022). Despite this, there are clear parallels between the adult and child literature.

The present study aims to understand more about how young people experience the goal setting and monitoring process, specifically using the GBO tool; and focusing on the helpful and unhelpful aspects of these processes, in the context of school‐based counselling. In doing so, it aims to provide suggestions for clinical practice and contribute to the development of more effective school‐based counselling evaluation practices.

## METHOD

The study was conducted within the context of a cluster randomised controlled trial (cRCT) which compared school‐based humanistic counselling plus weekly monitoring of personal goals (SBHC+G), with school‐based humanistic counselling only (SBHC). Young people who were in the SBHC+G group (*n* = 41) were eligible to be interviewed about their experience of setting and monitoring goals following the completion of counselling. This project received ethical approval from the University of Roehampton Research Ethics Committee (PSYC 17/262).

### Participants

A total of six schools were randomly allocated to the SBHC+G condition and six were randomly allocated to the SBHC group. All schools were state funded secondary schools (typical age range 11–18 years) located within the Greater London (UK) area. Of the 41 eligible participants from the six schools randomised to the SBHC+G group, 19 young people (46.3% of those eligible to participate) consented and were available for interview. Two schools did not respond to requests to facilitate interviews. Hence, the 19 participants included in the present study were recruited from across four schools (mean number of participants per school = 4.8, range 2–11 participants), each with their own counsellor. The majority of participants were female (*n* = 17, 89.5%), all were aged between 13 and 15 years (mean age = 13.7, *SD* = 0.7), and attended an average of 9.0 sessions (*SD* = 1.1) from a possible 10. Just over half (*n* = 10, 52.6%) were of white, European and/or British ethnicity, over two‐fifths (*n* = 8, 42.1%) were black, multiple, or another ethnic identity, and the ethnicity of the remaining participant (5.3%) was unknown.

### Measures

Young people completed the Outcome Rating Scale (ORS) at every session and the Young Person's Clinical Outcomes in Routine Evaluation (YP‐CORE) at baseline and final session. They also completed the Goal Based Outcome (GBO) tool at every session. The interviews were focused specifically on young people's experiences of setting and monitoring goals with the GBO tool.

#### Outcome rating scale (ORS)

The ORS is a four‐item self‐report visual analogue scale that aims to measure life functioning in the areas of personal well‐being, interpersonal well‐being (relationships with family, or other close relationships), social role (work, school and friendships), and overall well‐being (Miller et al., 2003). Respondents are asked to think about how well they have been doing in each area over the past week, indicated by making a mark on the 10 cm line provided for each item. Scores for each item range from 0 to 10, and total scores range from 0 to 40, with higher scores being indicative of higher levels of functioning. The ORS has been determined to have good levels of reliability and validity (Miller et al., 2003) and a clinical cut‐off of 28 has been determined for young people aged 13–17 years, with scores of 28 and below representing those in the clinical range (Sparks & Duncan, 2018). A reliable change index (RCI) of 6 points has recently been determined for the ORS (Redmayne et al., 2024). Five (26.3%) of the young people included in the present study showed no reliable change on ORS, 6 (31.6%) showed reliable improvement, 5 (26.3%) showed reliable deterioration, and 3 (15.8%) had missing ORS scores.

#### Young person's clinical outcomes in routine evaluation (YP‐CORE)

The YP‐CORE is a ten‐item self‐report measure of psychological distress (Twigg et al., 2009), which has been validated for use with 11–17 year olds. It asks young people how they have felt over the past week in response to items such as “I've been able to cope when things go wrong” and “it's been hard to go to sleep or stay asleep”. Each item is scored 0 (“not at all”) to 4 (“most or all of the time”), with inverse scoring for positively worded items. Total scores range from 0 to 40, with higher scores being indicative of higher levels of psychological distress. The measure has demonstrated good levels of reliability and validity and has established age‐ and gender‐specific RCIs and clinical bandings (Twigg et al., 2016). Ten (52.6%) of the young people included in the present study showed no reliable change on YP‐CORE, 6 (31.6%) showed reliable improvement, and 3 (15.8%) had missing YP‐CORE scores at end‐point, meaning that it was not possible to determine whether they made any reliable change.

#### Goal‐based outcome (GBO) tool

The GBO tool is an idiographic self‐report measure which allows the participant to set up to three personal goals for counselling using an open‐ended text box (Law & Jacob, 2015). Each goal is written on their own separate piece of A4 paper and below the written goal are 12 rows numbered 1–12 which each represent a session and where it is also possible to note the date of the session. As the present study only allowed up to ten sessions per participant, only rows 1–10 were used. On each row, there are the numbers zero (“not met at all”) to ten (“fully met”) which can then be circled by the young person or the counsellor. Progress on each individual goal can then be rated on this scale. A reliable change index (RCI) of three points has been established for the GBO tool in a school‐based setting (Duncan et al., 2023), meaning that change in either direction of at least three points can be considered “reliable” as opposed to fluctuations that might be expected as a result of measurement error. The GBO tool has demonstrated acceptable test–retest reliability, as well as acceptable convergent validity with standardised measures of psychological distress and emotional well‐being (Duncan et al., 2023).

Table 1 provides an overview of the goal categories and specific goals set by the young people in the present study. Seventeen young people had goals recorded, resulting in a total of 26 goals set (*M* = 1.53, *SD* = .72), with data on goals set missing for two of the young people. Ten of the 17 young people (58.8%) set one goal, five (29.4%) set two goals, and two (11.8%) set three goals. Furthermore, ten (52.6%) met the criteria for reliable improvement on personal goals, six (31.6%) met the criteria for no reliable change, and goal ratings were missing for the remaining three (15.8%) young people. None of the young people met the criteria for reliable deterioration on their personal goals.

**TABLE 1:** Goal categories and specific goals, with examples and frequency counts in relation to all goals set (*n* = 26) and by the total number of young people whose goals were known in the present study (*n* = 17).

Goal category	Specific goal	Example	All goals *n* (%)	Young people *n* (%)
Cognitive goals	Increasing/improving focus or concentration	“To be focused in class”	2 (7.7)	2 (11.8)
	Control over thoughts/actions (not specified)	“To be able to manage thoughts so they don't get in the way”	2 (7.7)	1 (5.9)
Emotional goals	Controlling or reducing anger	“To be able to manage my anger”	2 (7.7)	2 (11.8)
	Controlling or reducing emotions (not otherwise specified)	“To understand and cope with my feelings”	1 (3.8)	1 (5.9)
	Increasing happiness/reducing feelings of upset	“To feel less depressed”	1 (3.8)	1 (5.9)
	Reducing anxiety or worry/increasing calmness	“To have less anxiety”	2 (7.7)	2 (11.8)
Interpersonal goals	Dealing with bullying or being criticised	“To stand up for myself”	1 (3.8)	1 (5.9)
	Improving friendships and relationships (exc. Bullying)	“To reconnect with my friends”	3 (11.5)	3 (17.6)
	Talking more about feelings and experiences	“To be more open with people”	1 (3.8)	1 (5.9)
Goals targeting specific issues	Improving sleep	“To explore difficulty sleeping and look for ways to improve”	1 (3.8)	1 (5.9)
	Improving things at school/academic work	“Participate more in class”	1 (3.8)	1 (5.9)
	Other goals not otherwise specified	“To look after myself better”	2 (7.7)	2 (11.8)
Personal growth goals	Increasing self‐confidence or self‐acceptance	“Become more confident”	7 (26.9)	6 (35.3)

#### Semi‐structured interview schedule

A semi‐structured interview schedule (see Data S1) was developed which broadly focused on two questions: “What was your experience of setting goals?” and “What was your experience of monitoring goals?” Specifically, young people were asked about what they found helpful and unhelpful about these processes. Prompts were determined in advance to support the interviewer to elicit more in‐depth responses (for example, “Could you tell me more about that?”), however, the interviewer also asked questions that were not explicitly mentioned in the interview schedule, such as “how would you feel if you were able to fully meet that goal?”

### Intervention

All counsellors included in the trial had completed a core therapeutic training. This would have consisted, at a minimum, of a 1 year full‐time or 2 years' part‐time counselling qualification, plus a minimum of 100 supervised client hours. In addition, all counsellors had completed a minimum of 5 days' training in a specific manualised form of SBHC: an intervention based on the assumption that “distressed young people have the capacity to address their difficulties if they can explore them with an empathic, supportive and trustworthy counsellor” (Cooper et al., 2021; pp. 180–181). The intervention included using skills such as empathic reflecting, active listening, and encouraging open dialogue around needs and emotions. Counsellors also participated in a half day's training specifically on the use of the GBO tool, which included introducing it to young people, recording goals, and monitoring and reviewing goal progress. Counsellors were encouraged to support young people to set goals in the initial stages of counselling (within the first three sessions), and rate progress at every session using the GBO tool. The intervention also included the sessional use of the ORS (Miller et al., 2003) and pre‐ and post‐intervention use of the YP‐CORE (Twigg et al., 2009).

### Procedure

Participants were approached for interview following the last session of counselling via an appointed school counselling coordinator who was the lead contact between the school and the trial team. They were usually a member of the schools' pastoral care team. All young people and a parent/carer had provided written consent prior to involvement in the trial and all young people were asked for verbal consent prior to participating in the interview. Interviews took place between November 2017 and January 2019, and an average of 14.5 (*SD* = 19.7) days after the young persons' last attended session of counselling (range = 1–63 days).

Interviews lasted between 10 and 27 minutes, and were audio recorded. Recordings were then transcribed verbatim and anonymised prior to analysis.

### Qualitative analysis

All interviews, transcription and qualitative analyses were undertaken by the first author, an experienced researcher in the field of therapeutic interventions. This was supervised primarily by the second author, a practitioner who has extensive training and experience as a qualitative researcher and psychotherapist.

A “reflexive thematic analysis (TA)” was used which is “a method for identifying, analysing and reporting patterns (themes) within data. It minimally organizes and describes [the] data set in (rich) detail” (Braun & Clarke, 2006; p.79). Reflexive TA can be used to “provide a more detailed and nuanced account of one particular theme, or group of themes, within the data. This might relate to a specific question or area of interest within the data” (Braun & Clarke, 2006; p. 83). For example, in the present study the research questions were centred around “what are young people's experiences of setting goals in school‐based counselling” and “what are young people's experiences of monitoring goal progress in school‐based counselling” and the themes developed as responses to these questions. Data were explored semantically, affording importance to what young people said explicitly and working closely with the language they used. However, latent analysis was also important, for example when analysing the impact of goal setting on emotion, as emotions were often expressed more indirectly by participants (Braun & Clarke, 2022). Furthermore, a mix of inductive and deductive exploration was undertaken, where themes were both “data driven” (inductive) and “[used] existing theoretical concepts and literature [to] deepen the analytic interpretation” (deductive; Braun & Clarke, 2022; p. 27), such as qualitative findings from the existing literature around goal‐based practices (e.g. Di Malta et al., 2019; Penno et al., 2022).

The reflexive TA approach followed the six steps as set out by Braun and Clarke (2006) and consisted of: (a) becoming familiar with the data, (b) developing initial codes, (c) searching for themes, (d) reviewing themes, (e) defining and naming themes, and (f) writing a report. A more thorough discussion of the processes taken during these steps is available elsewhere (Braun & Clarke, 2022). Themes were initially developed by the first author and then reviewed and refined by the first and second author until full consensus was reached. Reflexive statements have been provided for each author, given that an essential component of this approach is to capture “the process of a researcher critically reflecting on their values, assumptions, expectations, choices and actions throughout the research process” (Braun & Clarke, 2022; p. 22).

At the end of each interview, the first author made an overall assessment of whether young people were generally positive, negative, or ambivalent about the goal setting and monitoring process. Those who were ambivalent generally gave mixed responses when they were asked about their experiences of the goal setting and monitoring process.

### Reflexive statements

The first author does not work therapeutically and has therefore has not used goals in therapeutic work. Prior to the commencement of her PhD research and, indeed, for the first couple of years of study, the first author believed, generally, that goals were a preferable means of measuring outcomes (as compared with nomothetic measures) due to their ability to allow the client to determine what a “good” outcome of therapy is for them. The fact that she is not practitioner who uses goals in therapeutic work enabled an openness to views that were more negative about the processes. Furthermore, the present study was grounded in critical realism (Bhaskar, 1975, 2008) and pragmatism, seeking to understand—within the boundaries of societal‐shaped understandings—the causes underpinning why some young people may find goal setting and monitoring “helpful” or “unhelpful”, as well as provide recommendations and implications for practice for counsellors.

The second author often uses goals in her work with clients but not with all clients, believing that some clients are not ready to set goals at the beginning of therapy/ or may (occasionally) be setting goals from a more “punitive place” within the self. She is curious about *when* goals are appropriate, galvanising, and for whom; and the best ways of monitoring these so that they do not eclipse other matters which might relate to the importance of sometimes being “stuck”, “lost” or not certain of a direction. She believes that young people may have a particular relationship to goals that is important to understand and differentiate from the findings with adult clients. Prior to the study, the second author had worked with some younger clients who were not keen to set goals and so had some expectation that goals may prompt ambivalent responses in the sample.

The third author has written extensively about goals from a clinical and psychological perspective and is a strong advocate of their therapeutic potential. However, he also believes that clients may differ in their experiences of, and attitudes towards, goal‐oriented practices; and that there is not a “one size fits all” best practice when using goal setting and goal monitoring in therapy.

## RESULTS

An overview of the themes and subthemes are presented in Table 2. Illustrative quotes for each theme are provided, accompanied by a pseudonym for each participant.

**TABLE 2:** Themes and subthemes.

Theme	Subtheme
Challenges in Goal Setting	Difficulty setting a goal
	Goals not memorable
	Goals can evolve
Complexity of Capturing Progress	Number didn't capture complexity/context
	Power of words in communicating goal progress
	Assigning a number to goal progress helpful
Impact on Motivation	Contributed to, or detracted from, a feeling of motivation
	Can mirror a feeling of “stuckness”
Preferences and Flexibility in Goal Monitoring	Weekly monitoring too often/every other week preferable
	Weekly monitoring preferable
	GBO tool helpful when used flexibly
Usefulness of the GBO Tool	Conversational tool
	Tangible representation of progress
	Increased insight
Focused the Therapeutic Work	
Shift in the Narrative About Goals	

Across the 19 interviews, young people's overall attitudes towards the goal setting and monitoring process were mixed. Eight (42.1%) were generally positive about the process, five (26.3%) were generally negative, and the remaining six (31.6%) gave ambivalent responses. Fisher's exact tests showed that there were no significant differences between attitudes towards the goal setting and monitoring process and whether a young person made reliable change on the GBO tool, ORS, or YP‐CORE, with all p‐values ranging between .71 and .96.

### Challenges in goal setting

Young people expressed several challenges that they encountered in the goal setting process, including difficulties setting a goal, goals not being memorable, and the evolving nature of goals.

#### Difficulty setting a goal

Several young people explicitly expressed difficulty in formulating a goal, highlighting that this is not a concept which is intuitive and natural for all young people. This may partly explain why a high proportion of young people set only one goal for counselling.

…he [the counsellor] had the form and he… said, “what… If you could pick one goal, what would you pick?” Or something along the lines of that. And I was kind of sat there and was like “ermmmmm”. (Rose)

In addition to the initial difficulty of setting a goal, some young people said that identifying a goal, when there may be many things they want to work on, can compound the difficulties. For instance, Katlyn said, “Setting specific goals was quite hard because it was kind of… trying to focus on three aspects of my life… when there's so many things.” Despite this difficultly with goal setting, it was also acknowledged by some as a beneficial exercise. Carly said, “It is a bit hard to think of goals…, but it was good to have them.”

#### Goals not memorable

Several young people struggled to recall the goals that they set for counselling. Phrases such as “I don't know” or “I can't remember” were common responses when asked if they could recall their goals and suggests that, for these young people, the goals, themselves, formed a small and potentially insignificant part of the therapeutic work. In other instances, young people could recall their goals, but it was evident from their response that they had struggled to remember them.

Erm we set… so… what did we say? Oh yeah… we need to be… more… participate more in class I think was one of them. (Katlyn)

This theme also reflected the composition of the interviews as a whole–where some young people spent most of the interview speaking about other aspects of the counselling that seemed to be more salient to them, such as the attitude of the counsellor.

#### Goals can evolve

Over the course of the therapeutic work, some young people described how the goals they set at the beginning of counselling became less of a focus as the therapeutic work progressed.

At the beginning it was a lot more to do with, like, my anxiety and that's what I thought I would end up talking about, but I ended up‐ we ended up talking about… things that had been going on at home and like, family stuff. (Carly)

Similarly, some young people said that new goals emerged over the course of counselling, emphasising the non‐static nature of goals.

Well, of course I still wanted to work on the goal of being able to like, focus in class… but, during the like, different sessions more… I guess, important things came up. That I would have like, rather… would have dealt with. (Sammy)

### Complexity of capturing progress

There were several instances where young people described the complexity of measuring goal progress using a number. In some instances, this was seen as a barrier, whereas in other cases this was seen as a helpful practice. Alongside this, young people made recommendations on how this could be improved.

#### Number didn't capture complexity/context

Assigning a number to goal progress was not always intuitive or easy for some young people, as noted by Rowena when she said, “I didn't really know where to scale it. Because I was like, ‘one is too like… bad’…and I wasn't sure if like, ten was ‘too good’ and five… I'm not sure‐ I wasn't sure about… numbers and like… what they scaled out as”. Several young people commented that this could be related to contextual factors. For example, some young people said that if something had happened prior to their session which had a negative impact on them this might be reflected in their ratings of their personal goals, introducing a bias to the rating process.

Maybe if I'd like fallen out with some people one week it would like “well, I haven't really been open with anyone… because I've now had an argument,” but like, there was some weeks where I'd be‐ have a really, really good week so then it would have gone up. So it was a bit like… it just had‐ depended on what had happened. (Maddie)

Similarly, assigning a number to goal progress was, at times, perceived to be somewhat reductive in that it did not account for fluctuations of emotions in different situations. For example, a young person who set a goal to “feel happier” reported a dilemma about how to capture this in a number when they had felt happier about one situation, but less happy about another:

Quite difficult because I might be then really happy about something but upset about another and… and I mean I just try and find an average but that can still be quite hard because that thing, even though the rest… of everything is fine… it's quite hard, that thing, that… just one thing has prob‐ puts… me down… a lot and I focus on it too much so… so it‐it was… it was hard in a sense. (Safaa)

This quote from Safaa also suggests that young people may find it difficult to assess situations evenly and may place more emphasis on one situation over another, which again has implications for the goal monitoring process.

#### Power of words in communicating goal progress

Closely linked with the theme “number doesn't capture complexity/context” was the recommendation from some young people that using words to communicate a sense of goal progress would be preferable to a number, to ensure that some of the complexity/context could be captured.

Rose It made sense but then I was just thinking, “I… don't know how to put it in a scale” because it I guess it's all just… different things and it's not like I can just pinpoint like, put it there and then like it's there‐ it's like… two or something. It was… I think it was more of like, “well, in some subjects I can concentrate”… “and some other ones I can't.” So I couldn't really… put that all… together and just put it down on a piece of paper.

INTERVIEWER OK… is there a way… that you think would be a better way of… rating goal progress… compared to a number?

Rose I think maybe just… writing like a sentence saying… like… “it‐I think… I've got more concentration in this lesson…‐whereas in this lesson I don't have as much concentration.”

Maybe a phrase like “I feel better than I did like, last week, about the goal,” or… something like that. Like, a number's very… final (Grace)

#### Assigning a number to goal progress helpful

However, not all young people felt that using a number to monitor goal progress was unhelpful. Indeed, a minority of young people valued assigning a number to goal progress and found it helpful to conceptualise progress in this way. Safaa said: “Well, I think it was nice to have things quantified and put into numbers.”

### Impact on motivation

Many young people noted that the goal setting and monitoring process could impact on motivation, both positively and negatively.

#### Contributed to, or detracted from, a feeling of motivation

Monitoring goal progress was often described by young people as contributing to, or detracting from, a feeling of motivation.

I would get like, happy and I'd think to myself, “OK, I'm making more progress”. But if I'm going down… if the line is going down… then, of course, it kind of… gets me depressed that I haven't made any progress. (Saffron)

Whilst goal monitoring was described as helpful in instances where clients perceived, or tangibly saw, that they were making progress towards their goal, for some young people, progress towards a goal becomes tied into their feeling of self‐worth. For instance, Nicole said, “[it] will show [inaudible] how up and down you feel and it kind of makes a person feel… like… ‘Ooooh look at me. I feel‐ like, I'm all over the place,’ and it's not really a good feeling.” This could contribute to a feeling that not making linear progress, or deteriorating, was unacceptable.

#### Can Mirror a feeling of “stuckness”

Both setting and monitoring personal goals reflected a feeling of “stuckness” for some young people. When setting goals, not knowing what they might want to change or work on could exacerbate this feeling.

I just struggle with that sort of thing because when someone says a goal… A goal I'm just like, “Well, what?” And then if they don't like, point out some stuff… then it, erm, then I'm not really going to get a goal to work, you know, towards or anything. (Riley)

Furthermore, one young person was explicit that, for them, if they were to have counselling again and found themselves setting the same personal goals, then that would highlight their inability to have previously achieved that goal and perhaps exacerbate a sense of failure:

I'd try other things if I have other problems… but like… because… if you write down the same goals every time then it wouldn't be actually helpful because… if you haven't done those things then that means you can't achieve those goals. (Will)

Similarly, not seeing any progress on goal ratings, or seeing a deterioration on goal progress, could reinforce this feeling:

I felt more like happier because I knew like I was getting somewhere but then like, when I felt the same I just felt like at least I'm not going down. (Naomi)

### Preferences and flexibility in goal monitoring

Many of the young people clearly articulated their preferences around the frequency and flexibility of goal monitoring, although there was not universal agreement on the best frequency for this. Some young people had a preference for weekly monitoring, whilst others would have preferred to do it less frequently.

#### Weekly monitoring too often/every other week preferable

Monitoring goal progress at every session was too often for some young people. Some young people suggested that not enough change was experienced within a one‐week period to make monitoring goal progress meaningful. Becky said “… sometimes it was a bit difficult because some weeks, like, nothing really happened”.

Others suggested that so much may have happened within the space of a week that scores could fluctuate greatly. This is perhaps more reflective of an immediate response to recent events, rather than real, sustained change, as illustrated by Maddie who said, “…it was a lot harder doing it every session because it would be like ‘well… either nothing's really changed, or like‐ it's like so much has happened’. So it was a bit like… well, it depended on what's happened.” In response to this, some young people suggested that monitoring them every other week would have been preferable, such as Becky who said, “so like, every other week. It would kind of… be a bit more… like, progress you could see.”

#### Weekly monitoring preferable

Conversely, some young people preferred reviewing goal progress weekly as a means of reminding them of their therapeutic journey. Edie said “I think it's OK to do it every week because it's like a weekly check in.”

Some young people said that the goals provided a consistent therapeutic element. This may then have provided some structure and predictability to the sessions.

I mean, her way was more comfortable for me because every like, every single counselling session… she tells me do you want to start with the points first, the goals? (Saffron)

I don't mind because then every week I would like, see how… I've made that goal and whether I've achieved it or not (Ama)

#### GBO tool helpful when used flexibly

Another helpful practice described by young people was the counsellor using the GBO tool flexibly. This included not imposing the measure on them at any particular time during a session and not pressuring the young person to complete it, but rather allowing the young person to make their own choice about if and when to complete it, allowing them a sense of ownership and control over the process.

She wouldn't make me do them, I did them because I wanted to and like, I could do them any time I wanted to do them. I never had to do them at the beginning or at the end… I could do them anytime. It wasn't something I had to… hurry up and get quickly done. (Nicole)

### Usefulness of the GBO tool

Young people indicated several ways in which the GBO tool was useful: as a conversational tool, as a tangible representation of progress and it's ability to increase insight.

#### Conversational tool

Using the GBO tool as a conversational tool was perceived to be a helpful practice for some young people by providing structure and opportunities for reflection and insight, when integrated into the therapeutic work.

And she goes through it. And then she explains to me, “so, you moved up. What makes you move up? You moved down. What makes you move down?” and actually explains… how I actually feel and then she like… gives me good advice and responds back nicely. (Saffron)

#### Tangible representation of progress

The GBO tool was perceived by young people as providing a tangible representation of their progress throughout counselling, rather than relying on memory alone, which brought a sense of “realness” in some cases. Lena said, “At the beginning I thought ‘I don't really know why we're doing this because I don't think it's going to help’. I don't know if it did help but… it was… nice because you then‐ as the weeks went on you could see how it was changing”. This was echoed by Rachel who said, “I feel… when I did that [rated goal progress] that I felt… it helped because like, I saw my progress, like, how I felt instead of like, thinking about it”.

#### Increased insight

For some young people, monitoring goal progress increased their insight into their emotions and brought some feelings into awareness that perhaps they were not conscious of without the deliberate act of rating goal progress. Hannah said, “I didn't feel anything but it made me realise like… how low my moods can be sometimes.” Edie said:

Yeah. So it's kind of‐ but then it also makes you think about really looking into it. And really feel like… so it's kind of like… pinpointing what you're feeling. Because sometimes people could be feeling something but then… when they really think about it, it's not as bad as they think… so it's kind of like… it's good but then it also‐ it's really difficult to kind of… because then you're admitting to yourself. Because if you are a low number then you're admitting that you're not OK.

### Focused the therapeutic work

Several young people commented that setting personal goals focused the therapeutic work. However, this was interpreted both positively and negatively for young people. For some, having some focus to the therapeutic work was beneficial as it provided some structure and boundaries to the sessions, as well as something to aspire towards.

It helps me because when I set my goals I was like “OK, I need to focus on how to make myself better or how to increase my friendships”. (Rachel)

I think, like, one step at a time. Like, I can't do everything at once, so I find it helpful, like, just having the one goal to just set my mind up to do it because if I had all these different types of goals then I would get lost on the way. (Hannah)

For other young people, having a focus appeared to limit the scope of the therapeutic work in a way which was unhelpful by not providing opportunities to talk about other aspects of their lives which they had perhaps not identified in their initial sessions.

I'd prefer not to do it because I like to talk about different things at different sessions like, depending on what's happened recently. And if like, by setting a goal then it kind of means you have to talk about that specific goal every single time. So I prefer like… not to set goals. (Sammy)

### Shift in the narrative about goals

A shift in the narrative about the goal setting and monitoring process was identified across some interviews. Here, young people may have initially spoken about the setting and monitoring of goals in a more negative or positive light, but their presentation around this changed as the interview progressed. This rendered it difficult to make an overall assessment of whether a young person held positive or negative views about goal setting and monitoring. An example of how the shift in narrative about goals was evident across an interview with Riley is provided below.

INTERVIEWER What did you think of being asked to rate your progress on a goal?

Riley Well, I didn't mind it personally. Erm… I know there were some people who did mind it, but personally I really don't mind it. It's like… so they know if they're actually getting anywhere with the sessions, if they need to change anything. So it's basically feedback for them, without actually having to say it entirely.

INTERVIEWER OK. So was that quite important to you? To be able to have it kind of visually down somewhere?

Riley Yeah

[*Towards the end of the interview*]

INTERVIEWER So, is there anything else at all you want to say? I mean, did you find setting a goal helpful? Did you find having a goal for counselling was useful or… or did you not particularly? [Laughs] There isn't a right or wrong answer.

Riley Erm, I didn't find it helpful.

## DISCUSSION

The present study builds on the existing research literature by exploring young people's perceptions and experiences of the goal setting and monitoring process using the GBO tool in the context of school‐based counselling. Several implications for practice are suggested which reflect the feedback gathered from young people.

The concept of goal setting may not be intuitive, or easy, for all young people, as reflected in the theme: “difficulty setting a goal”. It may also explain why over half of the young people (58.5%) chose to set only one goal for school counselling, despite having scope to set up to three. Previous research suggests that some of the factors associated with difficulties in goal setting in young people include the interference between the developmental need for independence and forming a collaborative relationship with an adult, low confidence, and/or feelings of hopelessness and previous negative experiences of working with goals in therapy (Feltham et al., 2018; Jacob et al., 2022; Kazdin, 2003). Indeed, over a third of the young people in the present study set a goal related to “increasing self‐confidence or self‐acceptance” which may go some way towards explaining this. It is possible that some young people may need more support from their counsellor to decide upon, and articulate, a goal for counselling. Indeed, the use of the word “goal” may have certain connotations for young people so framing goals as “wants”, “needs”, or “desires” may be more helpful to facilitate the goal setting process, if a client wants to do so. In addition, counsellors working with young people in this way may wish to explore the reasons behind why a young person has difficulty setting a goal and address these in a therapeutically sensitive manner. For example, if a young person has low self‐confidence then this could exacerbate a feeling of goal progress being tied into self‐worth, which is further discussed below. Future research, undertaken with larger samples which explore links between the types of goals young people set and their experiences of the goal setting and monitoring processes, may be warranted.

The findings from the present study suggest that those young people who experienced goals being used flexibly (e.g., allowing clients autonomy to choose if/when they rated goal progress, allowing new goals to be added as they emerged) found them to be more helpful, which aligns with existing research (Di Malta et al., 2019; Jacob et al., 2022). However, as counselling sessions were not recorded, it is not possible to determine the extent to which counsellors and/or clients in the present study explicitly referenced goals during the therapeutic work, or the extent to which they were being used flexibly. It is possible that what young people described as being helpful or unhelpful about the process was more about implementation than it was about the concept of goals. Future practice‐based research which utilises the recording of sessions may help to determine this. In addition, it seems reasonable to suggest that, as counsellors were aware that they were participating in a research project about the use of goals within school‐based counselling, they may have felt more inhibited to work flexibly with goals due to concerns of impacting negatively on the research. However, given that counsellors were not interviewed about their experiences of working with goals or their experience of participating in the research, this is a tentative interpretation.

In line with previous research, the present study provided some confirmation that helpful aspects of goal setting and monitoring, and use of I‐PROMs more generally, includes using the GBO tool as a visual representation of progress and enhancing a sense of motivation (Cairns et al., 2015). Our study also found parallels with the documented challenges associated with goal‐based practices, such as difficulties setting goals, enhancement of negative emotions when goals were not progressed towards, feelings of demotivation, or fear of failure (Di Malta et al., 2019; Penno et al., 2022). Taken together, this highlights the complex nature of individual differences: what may be helpful for one client may not be helpful for another. A “pluralistic” approach (i.e. focusing on the needs and preferences of the individual) to goal setting and monitoring seems to be indicated from the range of reactions and responses observed. Indeed, a prevalent theme which emerged from the present study, and which has been found in other samples (e.g. Di Malta et al., 2019), was the perception that goal setting and monitoring may enhance a feeling of “stuckness” and elicit negative feelings towards the self in young people who do not perceive themselves to be making progress.

There was no indication in the present study that attitudes towards the goal setting and monitoring process were linked with outcomes on the GBO tool, ORS, or YP‐CORE. It is important to note that the relatively small sample size may mean that it was underpowered to detect significant differences. A related point is that none of the young people included in the present study showed reliable deterioration on the GBO tool or YP‐CORE, highlighting a limited diversity of outcomes in the sample. Previous research with adult clients has found that those with more positive attitudes towards goal setting and monitoring show greater improvements on outcomes (Di Malta et al., 2019). We recommend that counsellors use their clinical judgement when working with goals, noticing when goal setting or monitoring might be exacerbating a condition of worth, or a feeling of “stuckness”, and adjusting their practice accordingly. For example, this might include holding the goal more lightly, seeing it as reflecting one part of self, or not setting and/or rating goals at all. Future research should also aim to gather feedback from a wider range of young people who have more diverse outcomes and look to determine what common characteristics there may be between young people who have more positive/negative experiences of working with goals to determine who it works best for. A strength of the present study was its diversity in terms of ethnicity, with over two‐fifths (42.1%) identified as being from marginalise ethnic groups. Hence, the findings may be considered to be of relevance to services working with adolescents from diverse ethnic communities.

Given that findings around the helpful and unhelpful aspects of goal setting and monitoring were mixed, we would advocate for young people being given a genuine choice in these processes. First, young people should be given an option of whether to set goals or not, and both of these options should be normalised. Practitioners may also want to explore these difficulties with the young person to understand the extent to which this may mirror their experiences outside of counselling to give attention to, and validate, what they need and want. Another recommendation for practice includes allowing the option of a narrative, written—or qualitative—explanation alongside, or instead of, numerical goal progress ratings to capture more of the context around goal progress. Finally, there should be more flexibility allowed around the frequency of goal progress ratings. In the present study, young people were asked to rate goal progress at every attended session—usually weekly—and this felt too frequent for some young people who did not feel that enough time had passed between ratings for there to be much change. However, these were not universal findings, and some young people did find it helpful to assign a number to goal progress at every session. Hence, these preferences should be checked with clients when working with goals and adjustments made. This aligns with the pluralistic framework which postulates that different clients may benefit from different in‐session activities at different points in time (Cooper & McLeod, 2011).

### Limitations

Whilst some limitations of the present study have already been acknowledged and discussed (i.e., a lack of diversity in terms of GBO outcomes), there are others which should be considered when interpreting the findings. A significant limitation of the present study was the high proportion (89.5%) of female participants. Largely this was due to the gender proportions from the cluster RCT (89.7% female) from which the sample for the present study was recruited whereas, more typically, females make up approximately 60%–66% of clients in school‐based counselling services (Cooper, 2013; Welsh Government, 2022). In part, this was also due to a significant proportion of participants (57.9%) being recruited from one single‐sex (all female) school. Although generally the findings from the present study align with those where the gender balance was more equal (e.g. Di Malta et al., 2019; Penno et al., 2022), caution should still be exercised if attempting to generalise the findings to all genders.

The young people included in the present study could be considered to be highly engaged in the therapeutic work given that they attended an average of 9 out of 10 sessions. Young people who attended fewer sessions may have had different experiences. It is, however, possible that goal‐focused work contributes to this engagement in counselling by enhancing the therapeutic alliance and helping young people to feel listened to, valued and understood, as has previously been postulated by Jacob et al. (2022). A further consideration is that counsellors' prior experience of working therapeutically with goals with young people was not considered or controlled for, which may have had an impact on implementation. This could, however, be considered a strength given that the sample interviewed for the present study would likely have experienced a range of therapeutic styles when working with goals, rather than a homogenous approach, which allows a nuanced picture to emerge when looking across the data set as a whole.

### CONCLUSION

The present study provides an important contribution to the literature by exploring what young people from diverse ethnic backgrounds have to say about their experiences of working with goals in counselling. Furthermore, it presents some suggestions for the advancement of working with goals therapeutically, namely that a bespoke approach to setting and monitoring goals is crucial in order to derive the greatest benefits and avoid counter‐therapeutic effects. However, further research capturing and auditing practice is required to understand more about what specific practices may enhance or detract from the goal setting and monitoring process, as well as to identify which clients it may work best for, and at what moments in the therapeutic process. Findings also indicate that goal setting and monitoring may not be a particularly salient aspect of the therapeutic process, as a whole, for young people receiving school based humanistic counselling, although it is not clear whether these findings extend to other therapeutic settings.

## AUTHOR CONTRIBUTIONS

**Charlie Duncan:** Conceptualization; investigation; writing – original draft; methodology; writing – review and editing; formal analysis; project administration; data curation; funding acquisition. **Jacqueline Hayes:** Conceptualization; methodology; formal analysis; writing – review and editing; supervision. **Mick Cooper:** Conceptualization; writing – review and editing; supervision; funding acquisition.

## CONFLICT OF INTEREST STATEMENT

There are no other conflicts of interest.

## Supporting information


Data S1.

## Data Availability

Research data are not shared.
