# Supplementary material for: Young people's experiences of setting and monitoring goals in school‐based counselling: A thematic analysis
Source: Psychol Psychother. 2025 Feb 24;98(3):663–82. doi: 10.1111/papt.12581 (PMC12346242; doi:10.1111/papt.12581)
Supplement: Supplementary file 1 — Data S1. [file PAPT-98-663-s001.docx]

**AGENCY: Qualitative Evaluation Topic Guide for Young People**

**Note for researcher: use this topic guide flexibly. Questions are written as a guide and researcher should use and probe as appropriate.**

- Introduce self, thank participant for time and speaking to you
- Go through information sheet and consent form again – reminder of purpose of research, confidentiality and reporting:
  - This research is being undertaken to understand more about how young people set goals for counselling and how counselling might help young people to meet those goals
  - I’m going to ask you to think back to when you set goals in your first session of counselling; what you thought of this and also what you thought of being asked to say how close you were to reaching your goals.
  - There are no right or wrong answers
  - If you want to stop at any point or don’t want to answer a question, then just say so
  - What you say to me during this interview will not be shared with teachers, other students, your parents or your counsellor unless you say something that makes me think that you or someone else is at risk of serious harm
  - Some of what you say may be used in reports and publications, but nobody will know it is you who said it
- Check permission to record before starting audio recorder:
  - The recording will only be accessed by the research team for the purpose of the research. It will be transcribed confidentially after the interview to allow analysis. The recording and transcript will be stored securely.
- Ask if they have any questions about what has been said or the interview
- Check that they happy to take part and for the interview to start.

*Section One: Background Information [approx. 5 minutes]*

1. Can you tell me about the counselling you received and what you thought of it?

*Section Two: Your experience of setting goals [approx. 10-15 minutes]*

1. Could you tell me about the goals that you set for counselling? [*If the young person struggles to remember their goals, read them out*].
2. What was is about these goals in particular that made you want to work towards them in counselling?
3. Looking back at those goals that you set at the beginning of counselling, how do you feel about them now? What do they mean to you?

*Section Three: Your experience of monitoring goals [approx. 10-15 minutes]*

1. You were asked to rate your goal progress during counselling [*insert condition here: at the beginning and end of counselling/at every session*]. What did you think of this?
2. Could you tell me a bit about how counselling helped (or didn’t help) you to work towards your goals?
3. Is there anything else you would like to tell me about your experience of setting and monitoring goals or counselling in general?

Close interview and thank participant.
